# Fourth of July Hazards: A Case Report on a Blast Injury to the Right Hand in a 21-Year-Old Male

**DOI:** 10.7759/cureus.70657

**Published:** 2024-10-01

**Authors:** Gina Saad, Damian Besada, Jenna Capobianco, Jessica Pagé, Nawaiz Ahmad

**Affiliations:** 1 General Surgery, St. George's University School of Medicine, Saint George, GRD; 2 General Surgery, St. Joseph's Regional Medical Center, Paterson, USA; 3 General Surgery, Wyckoff Heights Medical Center, Brooklyn, USA; 4 Plastic Surgery, NYC Health and Hospitals, Brooklyn, USA

**Keywords:** blast injury, firework injury, firework trauma, hand functionality, hand injury, hand rehabilitation, hand surgery, phantom limb pain, physical medicine and rehabilitation (pm&r), reconstructive surgery

## Abstract

Firework injuries pose a significant concern during celebrations, often resulting in severe trauma that can adversely affect an individual’s functional capacity and quality of life. The case highlights a young male who sustained an unwarranted incident after a malfunctioning pyrotechnic device detonated while held in his dominant hand. The patient underwent amputation of several digits while also salvaging the fifth digit and thumb, for which an open reduction and internal fixation were performed.

This case highlights the importance of understanding the extensive impact of these specific blast injuries. The complexity of these cases must include a multidisciplinary approach to treatment, which begins from initial treatment management to long-term rehabilitation.

## Introduction

Fireworks are widely used during holidays and cultural celebrations around the globe. The ease of accessibility and increasing popularity of consumer fireworks in the United States creates more risk for blast injuries. The most common blast injuries from firework handling are to the hand and may have long-lasting impacts on the individual’s function and quality of life [1]. 

Firework-related hand injuries encompass a range of trauma as well as injuries that require complex medical management and prolonged rehabilitation. There has been an increase in firework injuries from 2018 to 2022, with 65% of injuries about males [1]. Moreover, hands and fingers make up the largest percentage of estimated injuries [1].

The mechanisms of injuries sustained from firework-related explosions are manifold and include thermal, chemical, and mechanical forces. These blast injuries, often caused by the mishandling of fireworks, subject the handler to intense high forces that can result in severe laceration, tendon injuries, amputations, and/or fractures. The speed of the blast forces has the capacity of propelling debris significantly faster than a bullet [2]. The thermal forces, dependent on the hand’s proximity to the pyrotechnic device, can intensify the damage, especially due to its irregular shape [3]. Most firework injuries are sustained to the dominant hand at the distal phalanges as well as the distal interphalangeal joints [4]. Patients often experience damage to the thumb and index finger before they can release the device. This can cause the thumb to be hyperextended with palmar abduction, leading the first web space to be split [5].

Functional limitations resulting from firework-related injuries can be profound, leading to loss of motor strength, grip strength, and overall hand dexterity. Skeletal stability in the early stages helps reduce possible deformities and results in improved outcomes in return for function. 

These preventable cases often cause great surgical challenges due to the intricacy of the hand. It is also crucial to meticulously assess the extent of the injury and the viability of salvaging affected tissues. Evaluating the blast injury in this young adult male contributes to enhancing management efficacy and deepens the understanding of the complexity that blast injury withholds. In our case, we wanted to highlight that the injury which initially appeared to be unsalvageable, can be reconstructed to a certain extent in hopes of producing a functional hand when surgical intervention is performed early.

## Case presentation

**Figure 1:**
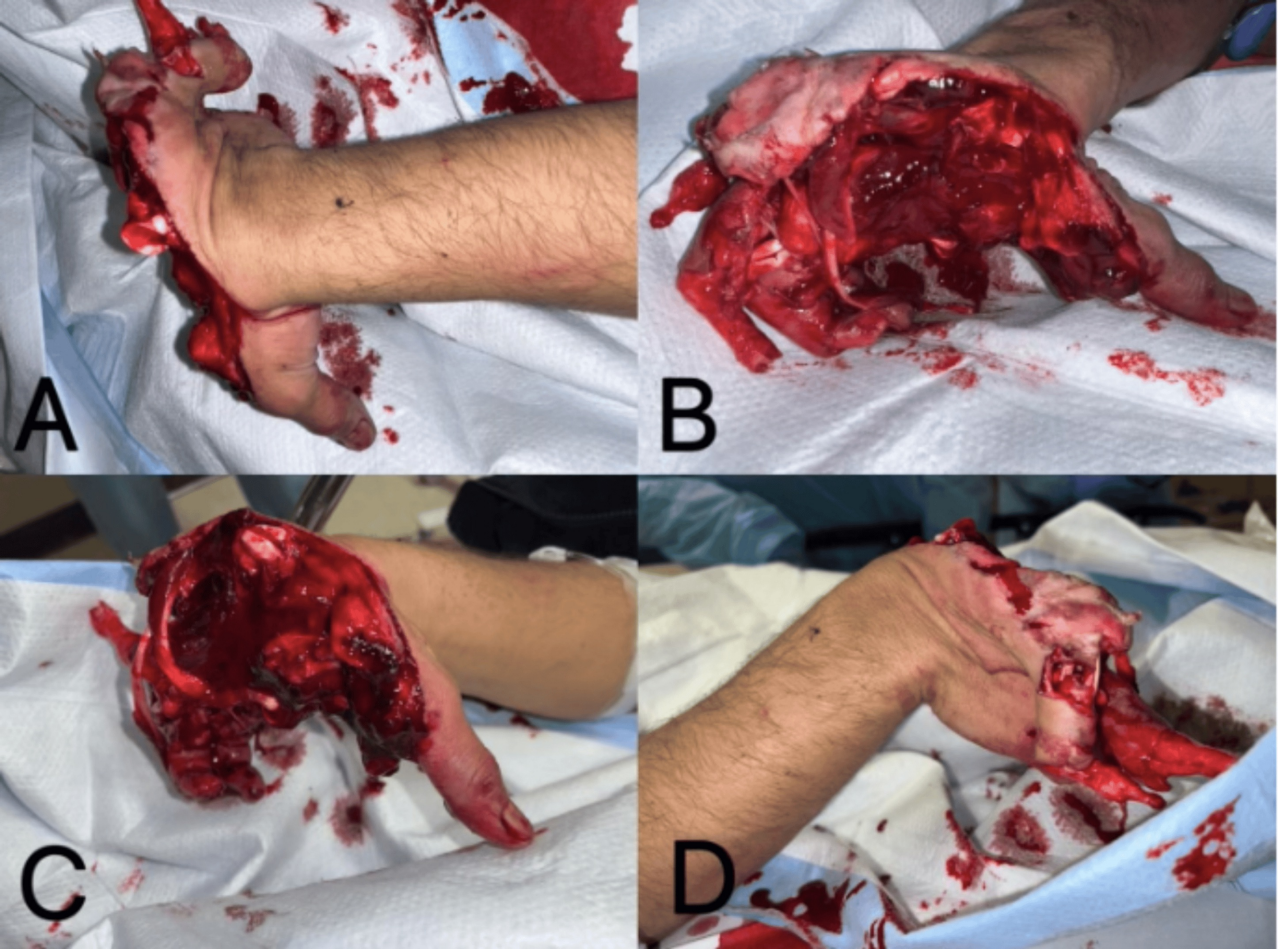
Hand at admission. The tendons of the second, third, and fourth digits, including the flexor digitorum profundus, flexor digitorum superficialis, extensor indicis proprius, extensor digitorum, and extensor indicis, were severely mangled (A, B, C, D). The thumb was dislocated and resting on the radial side (C). Significant damage was observed in the interosseous and lumbrical muscles (D). The carpal bones were exposed, and all other fingers were amputated by the blast.

The patient is a healthy 21-year-old, right-hand-dominant male who presented to the emergency department with a blast injury to his right hand sustained from a firework. The injury occurred when the patient attempted to adjust the firework after an initial failed detonation, resulting in an explosion while the device was still in his grasp. A thorough evaluation was conducted to assess the extent of the injury. A physical exam demonstrated a mangled extremity with multiple skeletonized digits and extensive tendon and muscular disruption. The blast resulted in splaying of the hand in half with multiple fractures, neurovascular compromise, and extensive soft tissue loss. The tendons of the second, third, and fourth digits, including the flexor digitorum profundous and superficialis, extensor indicis proprius, extendor digitorum, and extendor indicis, as well as the interosseous muscles and lumbrical muscles, were partially absent and otherwise irreparable. The remaining soft tissue of the right hand was edematous with profuse bleeding. The retained bony structures had haphazard alignment: the fifth metacarpal and phalanx were intact, but the first phalanx was dislocated and displaced radially. Carpal bones were exposed with visibly distorted association (Figure 1). The patient sustained no further traumatic injuries.

**Figure 2:**
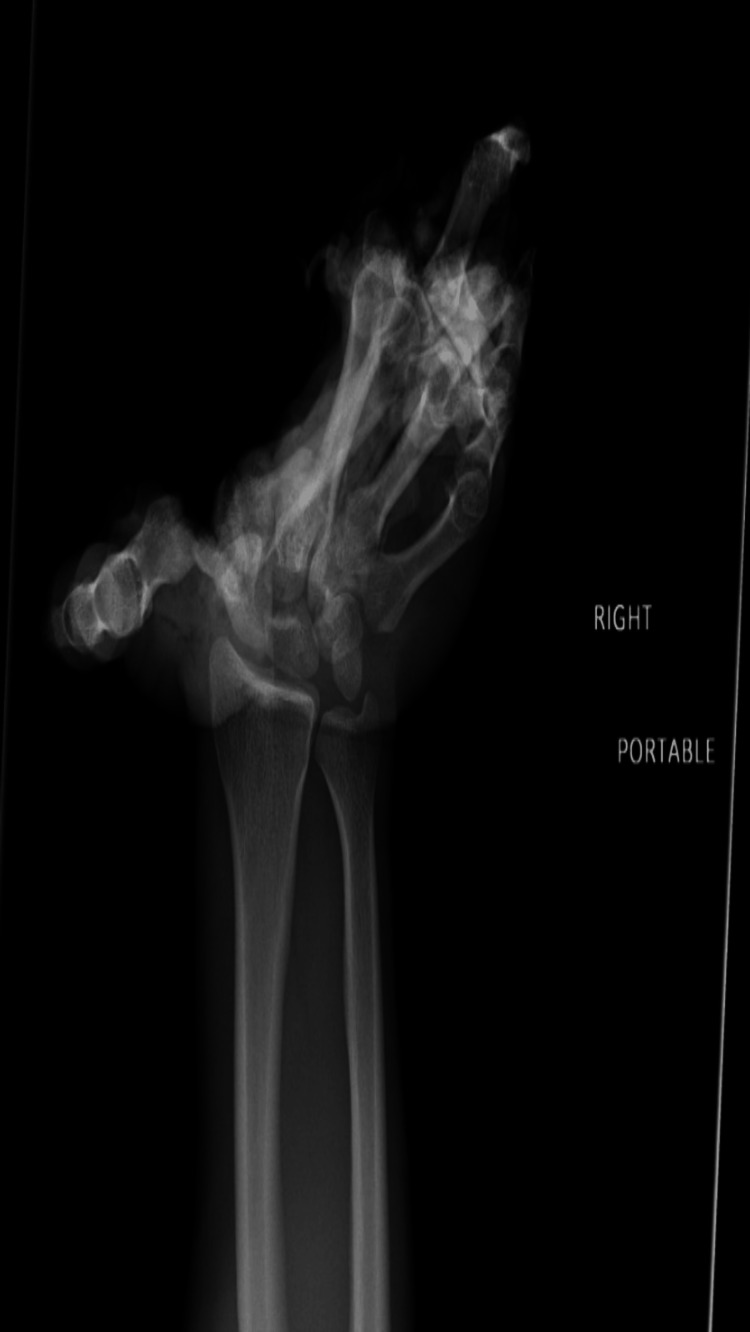
Radiographic image of the right hand on admission. An extreme valgus deformity of the first digit was observed. A dislocation at the base of the first metacarpal was suspected. Disruption of the distal carpal row and its corresponding articulations with the bases of the metacarpals was noted.

Radiograph images demonstrated extreme valgus deformity of the first digit. Dislocation at the base of the first metacarpal base was noted with disruption of the distal carpal row and corresponding articulations (Figure 2).

**Figure 3:**
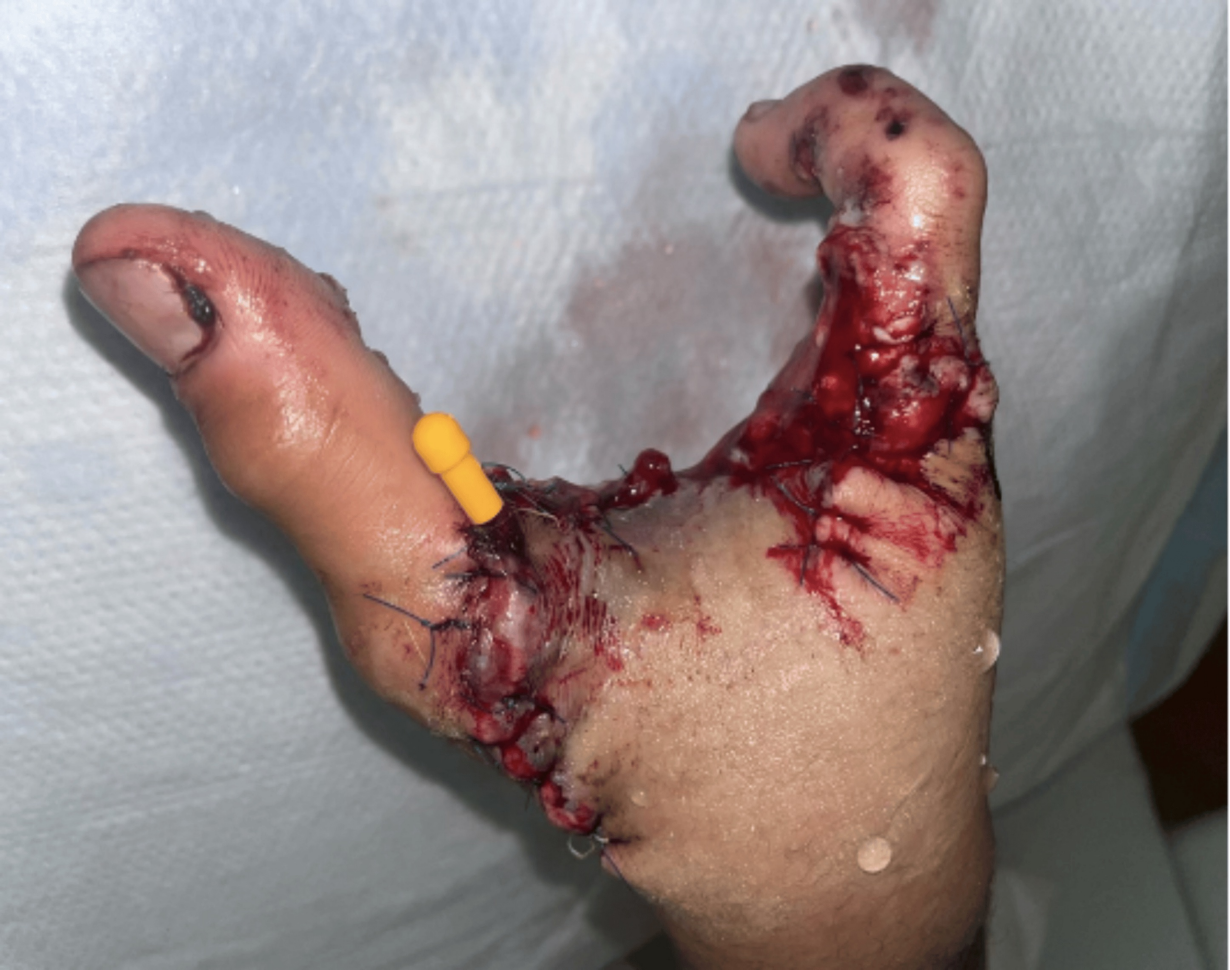
Postoperative image of the reconstructed right hand. Complex closure was performed using interrupted horizontal mattress stitches. The dislocation at the carpometacarpal joint was repaired with eight sutures, and Kirschner wires were drilled proximal to the joint.

**Figure 4:**
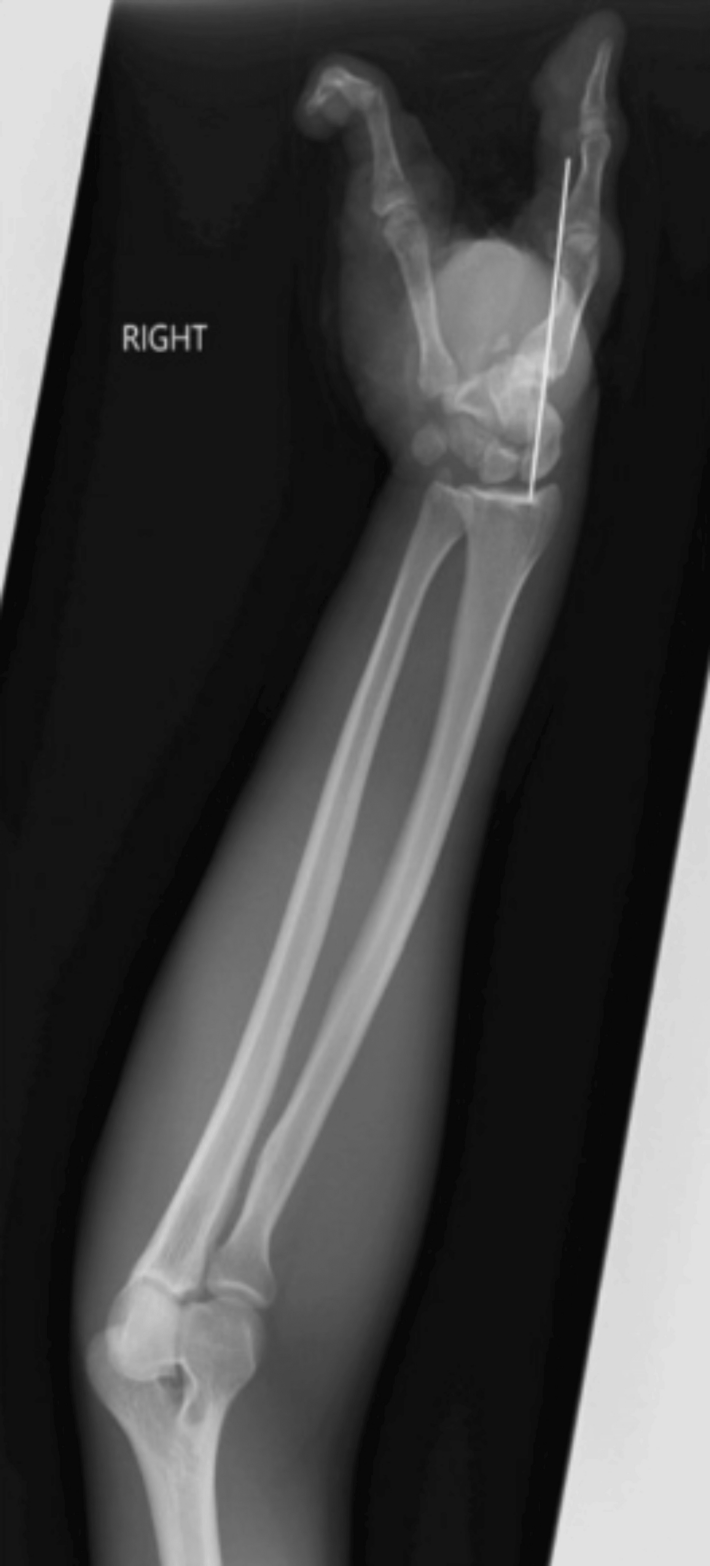
Postoperative radiographic image of the right hand. The X-ray image shows the salvaged digits and the outcome of the procedure, with stabilization achieved through the use of a Kirschner wire (K-wire) at the carpometacarpal joint.

The patient presented with acute distress due to pain and was afebrile, with his right hand wrapped in gauze. No additional deformities were observed elsewhere on his body. Physical examination suggested the potential for a complete hand amputation, given the extensive loss of tissue and total loss of function. After consenting to the possibility of losing his dominant hand, the patient was taken to the operating room after a 10-hour wait. Intraoperatively, the skeletonized second, third, and fourth digits were amputated at the carpometacarpal joint. Debridement of the ruptured extensor and flexor tendons was performed while preserving the dorsal flap for wound closure. The thumb was stabilized, and the hand was irrigated using pulsatile jet lavage. On the volar side, damaged tissue was debrided and bleeding was controlled through cauterization. A complex closure was achieved using interrupted horizontal mattress sutures. The dislocation of the thumb at the carpometacarpal joint was repaired with eight sutures, and Kirschner wires (K wires) were inserted proximally to enhance stability. Consequently, the thumb and fifth digit were preserved (Figures 3-4). A digital nerve block with 10 mL of bupivacaine hydrochlorothiazide was administered. The patient was also given intravenous broad-spectrum antibiotics (vancomycin 125 mL every eight hours and piperacillin-tazobactam 200 mL every six hours), along with analgesics and fluids.

Postoperatively, the patient exhibited paresthesias in the salvaged hand and intermittent phantom pain sensations in the amputated second through fourth digits. On postoperative day 2, the patient reported stiffness in the salvaged hand and limited wrist range of motion, which persisted throughout the hospital stay. A physiotherapist was consulted to facilitate rehabilitation and enhance mobility.

The patient was discharged on postoperative day 12 with a follow-up plan that included outpatient physiotherapy.

## Discussion

Firework injuries predominantly affect the hand and pose significant challenges to achieving successful reconstruction [1]. The extensive damage these injuries cause results in complex decision-making and necessitates meticulous planning for optimal outcomes. According to Ng et al., the typical pattern of blast hand injuries includes damage to the thenar muscle, dislocation of the carpometacarpal joint of the thumb, and finger disarticulation [6]. This case aligns closely with these patterns, as the injury is related to the grip strength primarily supported by the thumb, index finger, and ulnar fingers for stabilization. The close proximity of the pyrogenic device acts as a vector, with the most severe damage typically occurring on the palmar and radial aspects of the hand [5]. 

Given the complexity of blast injuries, successful reconstruction relies on key principles that enhance both the outcome and the functionality of the repair. These principles include debridement, revascularization, and bony stabilization [7]. The primary objective in this case was to preserve the right hand through debridement, ensuring a clean wound environment conducive to reconstruction and healing. For this patient, it was crucial to ensure adequate vascularization of the reconstructed thumb and fifth digit while maintaining alignment with the carpus to maximize the potential for functional rehabilitation. Kirschner wires (K-wires) were employed for the reduction and fixation of the thumb. In cases of extensive injury, revascularization may not always be feasible. Therefore, debridement and amputation are employed to facilitate adequate wound exposure, closure, and postoperative care [8]. Fractures of the hand frequently result in postoperative stiffness. During the wound healing process, type III collagen is deposited within joint capsules and tendons, potentially disrupting normal hand movement and leading to subsequent immobility [9]. To address the acute trauma experienced by this patient, it is essential to administer appropriate treatment to minimize joint stiffness and promote accelerated rehabilitation and functional recovery. Management of the hand injury should involve careful immobilization of the metacarpophalangeal joints (MCPs) in full flexion (70-90 degrees) while maintaining the interphalangeal joints in full extension. Additionally, the wrist should be positioned at 0 to 30 degrees of extension. This positioning helps stretch the collateral ligaments, reducing stiffness [10]. Although this approach is generally effective in mitigating post-traumatic stiffness, it is essential to tailor rehabilitation to the specific functionality and needs of each patient’s injury to achieve optimal outcomes.

Early hand rehabilitation following a blast injury is critical for optimizing recovery and preventing long-term complications. Postsurgical intervention is essential for promoting functional recovery and preventing the development of contractures. While a period of immobilization may be necessary to protect the hand from further damage, early intervention in cases of high-energy injuries is beneficial for improving the range of motion. Active-assisted motion exercises should be implemented early to enhance strength and improve lymphatic function. Prompt tendon mobilization exercises are also crucial for reducing the risk of adhesions following trauma, particularly by facilitating tendon gliding through their sheaths. Adhesions are more likely to develop at the proximal interphalangeal (PIP) joint than at the MCP, which can result in compensatory hyperextension at the MCP joint. Early intervention helps prevent these adhesions and minimizes long-term complications [11]. Additionally, a comprehensive and timely rehabilitation plan significantly impacts overall hand grip and controlled grasp [12]. Coupled with early surgical repair, early rehabilitation promotes mobility, reduces stiffness, and greatly enhances the likelihood of restoring normal hand function.

In this patient, who experienced paresthesias and tingling in the right hand following reconstruction, residual abnormal sensations are a common occurrence. A recent systematic review indicated that approximately 60% of individuals who have undergone amputation will experience phantom limb pain (PLP) [13]. Despite the absence of a physical limb, patients may report sensations of discomfort and tingling at the amputation site. This phenomenon is a critical consideration in surgical decision-making. PLP is a complex syndrome with an incompletely understood pathophysiology. Research using animal models has demonstrated that damage to soft tissues and nerves activates nociceptors, leading to localized inflammation. This inflammation can alter higher-order pain-carrying neurons and induce central nervous system reorganization. Both peripheral sensitization and central remodeling are key factors contributing to the persistence of PLP [14]. Aydın et al. found an association between the length of exposure to these inflammatory focuses and the frequency of developing PLP. Therefore, prompt amputation and debridement for high-risk patients with extensive injury, severe preoperative pain, and seemingly unsalvageable limbs is recommended as it removes the inflammatory focuses reducing the risk of developing PLP [15]. During the patient's admission, he experienced persistent paresthesia in the remaining two digits and intermittent pain originating from the sites of the amputated fingers, despite prompt debridement and amputation. Gaining a deeper understanding of the complexities and variations associated with PLP is crucial for developing a comprehensive treatment plan for patients who have undergone extensive trauma.

The management of hand trauma resulting from blast injuries necessitates an interdisciplinary approach to optimize rehabilitation and functionality. The complexity of the injury and the outcomes of reconstruction must be meticulously addressed and managed to achieve the best possible results.

## Conclusions

Firework injuries predominantly affect adolescents and young adults, necessitating a highly coordinated, interdisciplinary approach for optimal functionality and rehabilitation. The complexity of these injuries, as illustrated in the discussed case, is attributed to the intricate anatomy of the hand. The severity of blast-induced damage requires targeted surgical goals - debridement, revascularization, and bony stabilization - to facilitate hand preservation and lay the groundwork for successful reconstruction.

However, the path to recovery extends beyond surgical intervention. Postoperative care is critical, addressing challenges such as joint stiffness and the management of phantom limb pain and residual sensations in the amputated area. These complications underscore the necessity for comprehensive pain management strategies that address both physical and psychological aspects of recovery. 

This case underscores the significance of a patient-centered approach, emphasizing not only immediate physical repair but also long-term functionality and quality of life. The complexity of hand injuries demands adaptive rehabilitation and a thorough understanding of each individual’s unique needs. Integrating surgical expertise with a rehabilitation-focused strategy enhances the restoration process and maximizes functionality, ultimately improving the patient's quality of life and independence.
